# Essays on the Physiology of the Nervous System

**Published:** 1856-10

**Authors:** 


We are in receipt of "Essays on the Physiology of the Nervous system, with an appendix on Hydrophobia, by Benjamin Haskell, M. D., of Rockport, Mass.," a work of 87 pages, in pamphlet form, containing three essays and appendix. The character of the essays, and the style of the author may be learned from the following extracts. He says:

"I regard the nervous system as simple and uniform in its function, as in its structure; that, like all the rest of the bodily organs, it is by virtue of its physical properties, which are one and the same throughout all its homologous parts, it is of use in the body, and subserves the purposes of the mind. If ever it is proper to speak of vital properties, in relation to those activities by which its growth takes place, its integrity is maintained in health, and it is repaired in disease. But when it is formed, the nervous fibre, whether it is an attenuated cylinder containing fluid, or a solid, performs the same office wherever it is situated, whether in the brain, spinal marrow, nerve of sense or of motion. And that office is precisely what its particular structure adapts it to perform. Such is evidently the teaching of analogy, reason and common sense. The same is true of the vesicular portion; whether it forms a ganglion at the point where the nerves that go to an organ of sensation, meet with those that proceed to the muscles which move that organ, or occupies the centre of the spinal cord, or is distributed over the surface of the convolutions of the brain, we recognize nothing but an arrangement on physical principles by which arterial blood is brought into close relation with the nervous fibre; and whatever is the natural effect of the action of arterial blood as it passes to the venous state, (in all probability a dynamic effect,) on the nervous fibre, that we are bound, by all the rules of right reasoning, to consider the true and the only function of the vesicular portion. Nothing can be more absurd than to make a mere difference of form, where the same structure is brought into similar mechanical relations with another structure, a ground of difference in function, vital or otherwise.

"Of these physical properties of the nervous system, the mind avails itself, in establishing its relations of sensibility and motility with the body. And it does this, not by associating its faculty of sensibility as a whole, or its faculty of motility as a whole, with any part of the nervous system; but *particular* sensations and classes of sensations, *particular* motions and combinations of motions, are associated with the physical excitements of *particular* portions of the nervous system—a fact exemplified by the special senses, and the movements of respiration.

"These associations are incidental *to two fundamental and correlative laws or general principles*, which regulate the union of the mind and the body. One of these principles is mental, the other physical. The first is, that mind governs the motions of the body as directed by sensations. The second is, that the organ on which the impression, giving rise to the guiding sensation, is made, (if not in immediate juxtaposition,) is connected by nerve with the muscle to be contracted in order to move the organ.

"These two principles, which I think can be shown to be universal facts, constitute the key to the true physiology of the nervous system. With reference to the first of them, it is assumed that the action of the mind, neither in its sensitive nor voluntary department, is limited to those feelings or those volitions, which it stops to register in consciousness and thus remembers, but extends to all those phenomena, whose character of adaptation, and whose want of harmony with physical laws, prove them to be of the mechanism, or else to lie in a sphere remote from the penetration of our faculties, and one not given us to explore.

"The object of the union of the muscle to be contracted with the sensitive organ, appears to me to be, to enable the mind to avail itself of the physical property of the nerve whereby the two organs are brought, as it were, into apposition. Such would be the result, were we to suppose the physical quality of the nerve to be a power to receive and propagate minute impulses. Impressions from without would then be transmitted through the nerve and repeated on the muscular apparatus. And the attention of the mind, as held in sympathetic connection with the muscle, would be roused, just as it is, when an impression is made directly on the muscle, in any one of the peristaltic actions. All the forms of motion in the body are thus reduced to the same mechanism; and we are spared that contradiction of the law of parcimony,* which we make when we attribute one set of motions to the direct action of impressions, and another to influences generated in, and transmitted through, a nerve. In other words, the mind wills to feel at one and the same moment, the muscle that contracts, and the sensation under the direction of which it contracts; and it does this when the former is connected with the sensitive organ either by nerve or immediate contiguity.

"In like manner, it corresponds with the same view, to suppose the office of the ganglion or vesicular matter to be, to unite in one the nervous fibres passing from various surfaces of relation, as the muscles on one hand, and the organs of sense on the other. This may be conceived to be accomplished by the action of the arterial blood as it becomes deoxygenated, giving rise to minute impulses which excite a vibratory movement on the fibrils, and the interference of a new impression with which, is disseminated and felt throughout the whole mass. Thus the ganglion on the simple nervous cord may serve to unite the fibres that lead from the surface of touch of a segment, or small member, with the fibres that lead from the muscles that move that segment or member. The grey matter in the centre of the spinal marrow, unites contiguous segments and organs in associated relation, and that of the surface of the convolutions controls these minor centres, and unites in one all the nerves distributed to all the voluntary muscles and to all the organs of sense. Whether this be the true office of the blood in the grey matter, or not, one

* This term is happily assigned by Sir Wm. Hamilton to the old law, "that no more causes are to be introduced, than will account for the effects in philosophizing."

thing is pretty evident, viz: that the blood generates nothing, whether in the form of electricity, nervous power, or nervous influence, which is transmitted to the muscles. For when a nerve has been separated from its ganglionic connections some time, it will, when irritated, occasion contraction in the muscle to which it leads; a fact which proves that the ganglionic centre must act on some property already existing in the nerve, and affords nothing to the medullary fibre which it had not before.

"Thus, the conclusion we come to, is that while the nerve fibre connects the organs of sense with the muscle; the ganglion connects the different fibres from a number of points of a sensitive surface, with fibres leading to a number of muscles. Were there but a single organ of sense, and a single muscle to be contracted in consequence of sensations arising from impressions, on that organ of sense, the two would be united by a nerve without a ganglion. This conclusion, which establishes the uniformity of action of the nervous system, prepares the way for an investigation of the mode in which nature builds up the nervous system, and associates the powers of the mind with it."

Again, in comparing the views thus advocated by him, with those of Sir Charles Bell, he says:

"The common belief of vital properties of nerves, which this view opposes, derives its chief support from the authority of Sir Charles Bell, whose researches are well known; and a comparison of the principles just laid down with certain physiological and pathological phenomena, in order to show the manner in which these last are elucidated by them, would naturally lead us to advert to those of that writer. In the first place, then, I would state, that the conclusions of Sir Charles Bell never flowed legitimately from his premises. In his first experiments on the fifth nerve, before he had any theory to support, or rather before his theory had assumed a definite form, he drew the inference that this nerve was for motion. This, therefore, was the natural inference; and though subsequently, when on finding that it did not tally with those he drew from his experiments on the spinal marrow, he withdrew and reversed it, still there were residual phenomena, which threw serious doubts on its correctness in its amended form. The loss of all power in the lip, in an animal whose chief sense of touch resides in the lips, and the chief motions of which, would naturally be associated with it, the dropping of the mouth, and the drawing it to one side, seemed to indicate that something more than sensation was destroyed. Many labored attempts have been made to reconcile this contradiction by his followers. But they have not been successful. Contractions of the iris have also been produced by irritating the fifth; some distortion is produced by paralysis of that nerve; it sends fibres to muscles, and there are other signs of its agency in contracting certain muscles of the face, particularly the eyelids. Now all this is readily explicable on the supposition that the mind employs the fifth for touch, and those notions which it performs under the direction of touch.*

"Again, if we pass from the nerves of the face, to the spinal marrow, we find his experiments at once in conflict with those of Magendie and Bellingeri. While Sir Charles inferred that the anterior cords were for motion, and the posterior for sensation, Magendie inferred that the former were for motion chiefly, and the latter for sensation chiefly; and Bellingeri that the first were for the movements of flexion, and the last for those of extension. Scarcely anything deserving the name of an attempt to reconcile the results of those of the English, with those of the Italian physiologist, has been made. But since the discovery of the reflex function, Dr. Carpenter has endeavored, by the aid of special pleading and patchwork, to make the others coincide. By the reflex function, he explains, with some plausibility, how motions are produced, when, after section, the proximate ends of the posterior cords are irritated.† But when he explains how it is that sensation takes place through the anterior cord, he assumes the thing to be proved, and then makes use of it to prove itself. For he supposes that sensitive fibres from the posterior cord pass up from the point of union of the two cords towards the spinal marrow, not because they have been traced anatomically, but solely on

---

*For a full account of Sir Charles Bell's errors in relation to the fifth nerve, the reader may consult James O'Beirne's analytical correction of that writer's views respecting the nerves of the face, re-published, in this country, in the "Register and Library of Medical and Chirurgical Science," for 1834. Dr. O'Beirne comes to the conclusion, that either the fifth nerve must be allowed to have some other office than touch, or the motor portion must extend to branches of it, not now conceded by anatomists—which last alternative he adopts. Indeed it is the chief object of his essay to prove it. The view above given accords with the first. In one of the observations quoted by him, Sir Charles himself expressly admits "*a power of holding by the lips*, independent *of the seventh nerve*."

†The inconsequence of this conclusion is shown by an experiment of M. du Bois-Reymond. "If any motor nerve be selected which divaricates into two branches, (as, for example, the sciatic nerve of a frog, which divides above the bend of the knee into the tibial and peroneal branches,) and a galvanic stimulus be applied to either of these branches, this having been first divided above its insertion into the muscles, the electrotonic state will be developed, not merely in the portion of the trunk continuous with that branch, but also in that which is continuous with the other branch, as will be made apparent by the contraction in the muscles supplied by the latter." (*Carpenter's Physiology, Fifth American Edition*, pp 653–4) Here we have an undoubted instance of an irritation being transmitted through a nerve, when severed in part from its natural connections, in a manner opposite to the physiological mode of such transmission, as generally understood. Can any one say, that when the proximate ends of the posterior cords are irritated, the resultant motions through the anterior are not of an analogous character?

the ground that dividing the posterior cord puts a stop to the exhibition of sensation when the anterior is irritated. The true question is, whether there *are* any fibres either in the anterior or posterior cords by whose vital endowments sensation takes place. Is it not most natural to suppose, that irritation of the cut end of the anterior cord occasions convulsive or painful contractions of the muscles, and the connection being maintained by the posterior, the animal exhibits indications of suffering?"

He thus alludes to the "*corroboration of the views advanced:*"

"A further and somewhat singular confirmation of the idea above given of the functions of the anterior and posterior columns of the spinal cord, is derived from the following observations of Sir B. C. Brodie on injuries of that organ:

"The lower limbs are more frequently paralyzed than the upper, even when the lower part of the cervical spine has been injured. This circumstance is remarkable, as it is contrary to what happens when the functions of the spinal cord are interrupted in consequence of caries of the cervical vertebræ. In these last cases, the paralysis is often complete in the upper limbs for many weeks, or even months, before it extends to the lower. Paralysis of the upper limbs has been known to follow contusion of the dorsal vertebræ.

"These facts, which seem inexplicable on the common theory, are easily understood, when we consider that caries, affecting the bodies of the vertebræ, must involve the anterior columns some time before the posterior; whereas, in injuries, the processes are usually the first to suffer fracture or dislocation, and the posterior columns which are most concerned in the movements of the lower extremities, being nearly contiguous, will soonest feel the effects, and will therefore be most likely to be disturbed in their function.

"In No. 19 of Braithwaite's Retrospect, is an account of a discussion before the London Medical and Chirurgical Society, in which Marshall Hall took part, relative to a case in which there was palsy confined to the arms. The doctor was evidently at fault in his explanation of the case, simply because there was not room for it in his philosophy. According to his ideas, it was "almost impossible to imagine disease of the spinal marrow so situated as to induce paraplega of both superior extremities, without involving in its effects the parts situated below." After what has been said above,* it is unnecessary for me to enlarge upon such a case."

---

*On the connection between the motions of the arm and the specific senses, and through them with the cerebrum and anterior columns.

Again, on the causes which contribute to the reception of Bell's theory:

"The observations made in the two preceding divisions, enable us to estimate at their true value the experiments and reasonings of Sir Charles Bell, and the influence they have had on the subsequent progress of physiology. "The key to the system," says he, "will be found in the simple proposition, that each filament or track of nervous matter has its peculiar endowment, independently of the others which are bound up along with it; and that it continues to have the same endowment throughout its whole length." Here was his fundamental error. Long previous to his time, it had been suspected, from the occasional occurrence of paralysis of motion without loss of sensation, and the reverse, that different nerves were somehow subservient to these different functions. But the old physiologists who held this notion did not, as a general thing, any the less believe that both motion and sensation were functions of the mind, and not of the nerves. To him it was left to transfer, by a single stroke of his pen, these powers, from the province of the mind, and locate them in the nerves, as functions, springing from these imaginary vital endowments. And we look in vain in his works for any process of reasoning, grounded on physiological or psychological facts, to warrant the step. It was an assumption, neither more nor less; and it was an assumption, the necessity for which, it was incumbent on him to show, before he proceeded to experiment. Had he done this, his experiments would have been pertinent to prove *which* class of nerves were for motion, and *which* for sensation."

